# How independent is the international food information council from the food and beverage industry? A content analysis of internal industry documents

**DOI:** 10.1186/s12992-022-00884-8

**Published:** 2022-10-29

**Authors:** Daniel A. Zaltz, Lauren E. Bisi, Gary Ruskin, Connie Hoe

**Affiliations:** 1 Department of Health, Behavior and Society, Johns Hopkins Bloomberg School of Public Health, 615 N. Wolfe St, Baltimore, MD 21205 USA; 2 Department of International Health, Johns Hopkins Bloomberg School of Public Health, 615 N. Wolfe St, Baltimore, MD 21205 USA; 3 U.S. Right To Know, 4096 Piedmont Ave. #963, Oakland, CA 94611 USA

**Keywords:** International food information council, Industry documents, Conflicts of interest, Nutrition policy

## Abstract

**Background:**

The International Food Information Council (IFIC) and its partner foundation (IFIC Foundation) widely disseminate nutrition information and participate in relevant policymaking processes. Prior research has established a connection between IFIC and large food and beverage companies, representing a potential conflict of interest. The authors reviewed public records documents to investigate the connection between IFIC and industry, and to describe how IFIC communicates policy-relevant information about nutrition science to the public.

**Methods:**

The research team collected communications between IFIC and members of the research and policymaking communities by using state and federal transparency laws. The team analyzed the content of these documents with a commercial determinants of health framework while allowing for new themes to emerge, guided by the broad analytic questions of how and why does IFIC communicate nutrition information to policymakers and the broader public?

**Results:**

IFIC employs self-designed research and media outreach to disseminate nutrition information. Communications from IFIC and its affiliates related to nutrition information fell within major themes of manufacturing doubt and preference shaping.

**Conclusions:**

IFIC uses media outlets to preemptively counter information about the negative health impacts of added sugars and ultra-processed foods, and promotes a personal-responsibility narrative about dietary intake and health. IFIC and its affiliates disseminate a narrow subset of nutrition and health information consistent with corporate interests and in opposition to public health policies associated with improved population health.

**Supplementary Information:**

The online version contains supplementary material available at 10.1186/s12992-022-00884-8.

## Background

Opposition to public health interventions targeting the consumption of unhealthy foods and beverages is largely concentrated within industries that produce and distribute these unhealthy products, which include sugar-sweetened beverages (SSBs) and other ultra-processed foods [1–6]. Accordingly, a growth in the examination of the commercial or corporate determinants of health [7–9] has fueled critical appraisal of industry action to oppose these interventions [10–12]. For example, researchers have noted conflicts of interest in large studies funded by transnational food and beverage companies for their a priori biases towards producing research narratives sympathetic to corporate interests [13]. Others have reported systematic biases in public health nutrition studies based on funding sources, noting that those funded by large food and beverage companies may be up to five times more likely to report findings sympathetic to business interests [14, 15]. Still, funding academic research is only one of many tactics used by these companies to promote their profit interests over the public’s health.

In addition to direct funding of scientific research, large food and beverage companies have used media, communications, and political lobbying as synergistic strategies to oppose evidence linking consumption of unhealthy foods and beverages to increased risks of chronic diseases [13, 16, 17]. For example, studies have found that companies wield their economic and political capital in an attempt to influence national dietary guidelines and policies designed to impact large populations, often using non-profit organizations which they fund as stakeholders in influential media campaigns [3, 13, 17, 18]. These influence campaigns are typically designed to reframe public narratives related to diet and obesity as problems of individual choice, versus structural or environmental factors, thereby focusing efforts away from broad population-level, evidence-based interventions [19–23].

Prior studies have made use of publicly-available documents to analyze mechanisms through which large companies influence research and policy via industry front-groups, with many focused on the food and beverage industry-funded International Life Sciences Institute (ILSI) [3–6]. A front group is “an organization that purports to represent one agenda while in reality it serves some other party or interest whose sponsorship is hidden or rarely mentioned.” [24] Front groups are an example of a third-party technique, which is considered the ‘heart of public relations’ as it can provide controversial actors with a legitimate voice [25]. Prior research has also shed light on the connections between ILSI and other organizations that may act as stakeholders in the opposition to public health interventions designed to reduce consumption of less healthy products [4]. Recent document analyses highlight the International Food and Information Council (IFIC) as a “sister organization” established alongside ILSI to act as a media and communications group [4]. The official stated mission of IFIC, which is a non-profit organization, is to “effectively communicate science-based information about health, nutrition, food safety and agriculture.” [26] The organization is primarily funded by food, beverage, and agricultural companies [26]. Despite the close connection between ILSI and IFIC, little is known about the how and if IFIC acts on behalf of its funders to oppose public health dietary interventions. Crucially, while industry front-groups may engage in tactics to influence media and public discourse related to food and nutrition, little is known about how these groups specifically engage in such activity. The purpose of this study was to examine how IFIC generates and disseminates nutrition information to policy stakeholders and the general public.

## Methods

### Overview

This was a qualitative analysis of publicly-available documents and email communications between IFIC leadership and key board members and associates in across various academic disciplines. These documents were accessed via state and federal transparency laws. This study was considered not human subjects research and therefore exempt from review by the Institutional Review Board of the Johns Hopkins Bloomberg School of Public Health.

### Analytic approach

The research team organized this study into five phases. First, the team used readily-available information to build a descriptive profile of the organization and stated mission of IFIC. This phase included a review of IFIC website materials, documents published under the IFIC heading, and public tax documents. Second, the team retrieved study documents via US state and federal transparency laws. Third, the team read through all documents in totality to gain an overall understanding of their contents. Fourth, the team summarized consistent concepts throughout the documents via an integrated approach to develop and apply thematic codes to these documents [27]. Specifically, the team used a framework by Lima and Galea (2018) to deductively code the documents [28]while additionally using an inductive process to allow for new themes to emerge from the documents and in discussion across the research team. The framework includes five “vehicles of power” and their component “practices of power,” which operationalize specific actions taken by corporations and their affiliates that impact broad social determinants of health and lead to population health disparities. There are 27 total “practices of power,” all of which we used as themes to deductively code the documents [28]. We adapted the description of these 27 practices to pertain specifically to nutrition research and policymaking in order to align with the official remit of IFIC (Additional file 1). Finally, the team synthesized the findings from this integrated approach and summarized the overall findings based on consistent themes identified throughout the documents.

### Data sources

U.S. Right To Know (USRTK), a non-profit investigative public health research group, made requests under the Freedom of Information Act (FOIA) [29], Hawai’i Uniform Information Practice Act, New Mexico Inspection of Public Records Act, Illinois Freedom of Information Act, California Public Records Act, West Virginia Freedom of Information Act, Colorado Open Records Act, Minnesota Government Data Practices Act for email communication between Carol Boushey, Lowell Catlett, Bruce Chassy, Bob Goldberg, Gregory Hand, James Hill, Mark Kern, Joanne Slavin, Joanne Spahn, and Alison van Eenennaam and IFIC affiliates between 2012 and 2018. These individuals were identified as having ongoing influential roles with or connections to IFIC based on publicly-available information related to IFIC board positions and media affiliations. The research team additionally reviewed publicly-available tax records for IFIC and the IFIC foundation from fiscal years 2011–2018 [30, 31], and reviewed one document mentioning IFIC that emerged from recent litigation against the agrochemical company Monsanto (now owned by Bayer). The research team received all data in .pdf format and arranged these files based on the individual to whom they were attributed from the document requests. All of these documents consisted of email communications and attachments, with some personal information (e.g., cell-phone numbers, birthdates) redacted by the agency from which they were received.

### Document review

The documents were first reviewed by USRTK. Then, the research team randomly chose 10% of all documents to code in triplicate. The team used this initial screening as a method to calibrate the use of the thematic codes. At the end of this initial coding the research team met and discussed all codes and notes, using a majority consensus to decide which codes pertained to different sections of text. Next, the team randomly divided all files among two study team members (DAZ, CH) for full-text review. A third reviewer (LB) recoded a random sample of 10% of all documents. All reviewers met to discuss which codes applied to each section of the documents, and when discrepancies existed (< 5% of all coded sections), reconciled those differences via majority consensus within the entire research team. The team met bi-monthly to discuss current progress, reconcile differences in codes, and refine the review process. The research team conducted all analyses using MAXQDA 2020 software (VERBI Software, 2019).

## Results

### Summary of the international food information council

IFIC is a trade association focused on communicating scientific evidence related to nutrition, agriculture, and health to policymakers and the general public [26]. IFIC broadly disseminates nutrition information via partnerships with institutions in academia, government, and multimedia outlets. For example, IFIC conducts an annual survey of consumer food preferences, and partners with academic and professional organizations to disseminate nutrition information to dietitians and other health professionals [32, 33] According to its website, the IFIC Board of Trustees is made up of a majority of “public academic researchers and experts in food science, nutrition and agriculture,” and further states that it does not engage in any political lobbying advocacy for any specific business interest, but rather promotes “science-based information on nutrition, food safety and agriculture” [26].

IFIC is further split into organizations: IFIC and the IFIC Foundation, both of which are tax-exempt organizations under the 501(c) subsection of the US Internal Revenue Code [30, 31]. IFIC is a 501(c) (6) organization,), often referred to as a trade association, which is defined as an “association of persons having some common business interest, the purpose of which is to promote such common interest.” [34] Organizations designated as tax exempt under subsection 501(c) (6) may not engage in activities which are directly for-profit (e.g., selling goods or services), but may be “devoted to improving business conditions of one or more lines of businesses,” and may engage in certain forms of political lobbying [34]. The IFIC Foundation, on the other hand, is a charitable 501(c)3 organization. All published materials on the official IFIC website (www.ific.org) state that it is a 501(c)3 organization, and therefore presumably refers to the IFIC Foundation. However, the core leadership team, including CEO, is shared between IFIC and the IFIC Foundation [30, 31], and due to this sharing it is usually hard to discern whether any given action is emanating from IFIC or the IFIC Foundation. Publicly available federal tax documents (IRS forms 990) for IFIC and the IFIC Foundation include specific membership contributions but do not disclose the name of each contributor [30, 31]. However, documents collected in this study show that during fiscal year 2013, the IFIC Foundation received contributions from nine sources: eight large food and beverage companies (Coca-Cola, General Mills, Hershey, Kraft, Nestle, PepsiCo, Unilever, Kellogg) and the United States Department of Agriculture (USDA)).). Between 2004 and 2018, approximately 80–90% of IFIC total revenue, which ranged from approximately $3.4 M - $5.2 M per year (Additional file 3), came from program services that are comprised almost entirely of membership dues [30].

### Overview of thematic analysis

In total, the data comprised 29,252 pages of email communications and attachments spanning years 2012–2018. The research team identified three major themes to emerge from this document review. The first and most prevalent theme identified was preference shaping, which refers to communications designed to promote specific beliefs about nutrition and health. The second theme identified by the research team as consistent and prevalent throughout these documents was manufacturing doubt, defined as the use of specific evidence and rhetoric to create doubt about negative health impacts of specific foods or food groups. Finally, the study team identified as a prevalent theme the consistent use of self-funded research disseminated by key opinion leaders in academia and government positions.

### Preference shaping

Nearly all coded sections within the data fell under the “preference shaping” code, which includes the use of key opinion leaders and multimedia communications to promote narratives sympathetic to business interests [28]. IFIC appeared to engage in a variety of preference shaping tactics leveraged through close connections with for-profit food and beverage companies. In a newsletter sent to its members and affiliates on February 6, 2017 (Additional file 2), IFIC leadership announced changes to the Board of the IFIC Foundation and included the following summary statement:

The majority of the board, which oversees the IFIC Foundation, comprises representatives from universities, governmental bodies, research laboratories, and public foundations. The balance of the trustees represent [sic] for-profit companies.

Emails between IFIC leadership and its Board of Trustees revealed the connection between IFIC programs and its member contributions. In 2014, then CEO Dave Schmidt sent an email to the Board of Trustees containing a summary of *Understanding Our Food*, a food and nutrition education campaign designed by IFIC. The summary defined the education campaign as follows:

To communicate the important roles of modern food production, processing, and technology in providing a safe, affordable, and nutritious food supply.

Later, Schmidt commented on the goals of the program:

Since its inception, the initiative has been working to impact consumers’ perception of processed foods through science-based information while also recognizing consumers’ emotional relationship with food.

Schmidt continued, commenting on the program impact:

The creation and distribution of the *Understanding Our Food* Tool Kit, participation in the Food and Nutrition Science Solutions Task Force, and development of the Alliance to Feed the Future are just a few of the successes the initiative has achieved.

Schmidt then commented on program funding:

We are looking forward to building upon these successes in the coming years and appreciate financial support from several member companies.

The email then contained a list of all contributors, which are comprised entirely of food, beverage, and agro-chemical corporations (Additional file 4).

Schmidt then appeared to solicit voluntary contributions:

From previous research we know that negative perceptions of processed foods are deeply rooted and cut across all consumer demographics. For this reason, it is crucial that the benefits of food processing are communicated by credible individuals and organizations such as the IFIC Foundation. If you are not a current supported, please consider contributing the suggested $10,000 voluntary contribution to the Foundation to support the *Understanding Our Food* initiative to health further our work.

### Manufacturing doubt

IFIC organized meetings between media outlets and selected scientific researchers covering specific food and nutrition topics. For example, in 2013, Kris Sollid, then Associate Director of Nutrients at IFIC, emailed Dr. Mark Kern from San Diego State University:

This August, we will be hosting a Media Briefing in New York City and would be honored to have you speak at the event. The Media Briefing is with New York-based editors, journalists, influential bloggers and registered dietitians in the media. The event will focus on communicating the scientific evidence on carbohydrates and sugars: what it suggests and equally important, what it does not suggest; impact on health; and role in a healthful diet.

Sollid later mentioned the history of these events:

We’ve done similar briefings in NYC over the past few years and have found them to be very effective.

Sollid included a description of IFIC compensation policy:

As part of our mission, our commitment to science and amplifying the voices of credential [sic] and respected experts such as yourself prohibits us from paying fees for speaking to media or approving your comments. We are, however, able to cover travel-related expenses and will discuss those with you in more detail in a follow up email.

Kern later sent a copy of his presentation to be made at this media briefing, titled “The Sweet Truth: Unraveling the Myths and Mysteries of Sugars.” The presentation appeared to downplay the relation between sugar consumption and negative health outcomes and cites a variety of empirical studies and reviews related to sugar consumption, sources of sugar, and their impacts on health (Additional file 5). The majority of these studies cited in this presentation were funded by food and beverage companies or sugar manufacturers, and some of the conclusions provided by the authors of the studies cited by Kern were contemporaneously refuted by large bodies of evidence [35–38].

### Media influence and preemption

Another central theme in this study was the proactive use of media to preempt specific messages in the public conversation related to nutrition and health. In a 2013 email to the IFIC Board of Directors and the IFIC Foundation Trustees, then Executive Director Kimberly Reed detailed plans to provide an early critique of two books related to the health impacts of processed foods:

In anticipation of the Feb. 26 release of the books *Salt, Sugar, Fat* by Michael Moss and *Pandora’s Lunchbox* by Melanie Warner, we wanted to update you on recent media coverage and actions that we are taking.

The email then summarized tactics to be implemented by IFIC and the IFIC Foundation:

Based on Moss’ just-published adaptation, we are:

- Moving up the Feb. 27 release of the Feb. edition of our *Food Insight Newsletter* that features our book reviews. These reviews will also be repurposed as blogs on foodinsight.org
- Providing general and committee-specific talking points in the near future.
- Exploring additional options to enhance our engagement in the digital media measured by the extent of coverage.

IFIC described a media strategy, called the “Media Dialogue Program,” which is defined as follows in their 2013 IRS form 990:

To pursue aggressive, ongoing media relationship building to provide context and improve accuracy in the reporting of food safety and nutrition issues, as well as to support long-term educational outreach to journalists and experts.

IFIC pursued this “educational outreach to journalists” through what appears to be informal agreements with academic researchers. In a 2014 email, then Associate Director of Nutrients, Kris Sollid, emailed Dr. Mark Kern from San Diego State University regarding the documentary “Fed Up” by Katie Couric:

Just wanted to let you know that we did issue a release to media that included a list of available experts for comment – you were included per your agreement. Have you had any contact, by chance?

Sollid included in this email exchange a summary of the movie which contained the following note:

IFIC member companies’ specific products and programs featured were Coca-Cola, PepsiCo, McDonald’s, General Mills, Kellogg’s, Pizza Hut/Taco Bell/KFC, and Kraft Foods.

Later in the email exchange, Sollid asked Kern to submit a media statement in anticipation of media coverage of Couric’s documentary:

Also, we’ve just been contacted regarding a show that will air tomorrow (Katie Couric Show) on sugar with Dr. [Robert] Lustig as guest. Although they are not seeking an additional live guest, they are potentially looking for a statement from a leading researcher that may offer a different perspective than Dr. Lustig. In advance of any potential submission of an expert statement, I was curious if would you be willing/able to submit a scientific statement on the topic of sugars, HFCS, fructose, etc.? If so, to limit your time-burden, we could do the “heaving lifting,” so-to-speak, and utilize your scientific breakdown of Dr. Lustig’s “Fat Chance” to draft a quote or statement for your review and approval.

In this particular instance, the statement discussed above was not included in the media coverage. However, the interaction between IFIC Foundation and scientific researchers in anticipation of media coverage that may be detrimental to the food and beverage industry was observed multiple times throughout this document review.

In another example of media influence, in 2015, then Senior Director of Communications Matt Raymond sent an internal email regarding recent efforts by IFIC to respond to an editorial in the *British Journal of Sports Medicine* that challenged the connection between physical activity and obesity [39]:

We were contacted prior to the article’s release by Food Navigator. We reached out to several experts and connected four of them with Food Navigator, three of whom (including Marianne Smith Edge) were quoted in the story, providing the kind of balance not often seen when negative journal articles are first published.

The media outlet Food Navigator mentioned above described itself as the “leading online news source for the food industry” (foodnavigator.com). When this email was sent, Marianne Smith Edge was the Senior Vice President of Nutrition and Food Safety at IFIC.

### Supporting research and key opinion leaders

IFIC funds and implements an annual survey of US consumer food preferences, and publishes results from this survey in academic journals [40]. Internal summaries of the survey, sent from IFIC to their Board of Trustees and a variety of academic advisors, included the following summary point:

It appears that poor eating in more a matter of lacking will than knowledge – adults appear to know the healthfulness of their eating habits.

This framing appeared throughout the summary document, including the following commentary:

Being more thoughtful about the amount one consumes and planning consumption occasions leads to healthier choices and are behaviors more commonly observed among healthy weight compared to obese persons.

The internal summary of the survey additionally contained a timeline describing IFIC’s dissemination strategy:

1. Outreach to secure lead author for manuscript
2. Hold stakeholder call to review complete findings and discuss promotion of findings
3. Manuscript submitted to peer-reviewed publication
4. Continued promotion of findings, including peer-reviewed publication; submission of comments to 2015 Dietary Guidelines Advisory Committee; presentations at relevant, appropriate opinion leader and stakeholder annual meetings and conferences

Throughout the documents there was a consistent focus on behavioral determinants of dietary intake. In the IFIC 2012–2015 Strategy Document (Additional file 6), one stated goal was:

The Foundation will be recognized by key stakeholders as a credible convener on consumer attitudes and behavior related to food safety and nutrition and their role in health promotion and disease risk reduction, including non-communicable diseases.

One objective stated beneath this goal was:

Elevate value and significant insights of the *Food and Healthy Survey* to better inform strategic initiatives with behavioral focus.

One way IFIC appeared to elevate the public-facing value of their research and conclusions is through spokespeople and key opinion leaders. A staff member (Joanne Spahn) at the Center for Nutrition Policy and Promotion, USDA, for example, served on the Academic Advisory Board to support IFIC in the development of DataDish - a checklist that could be used to evaluate research articles. She was subsequently invited to participate on a 2017 FNCE panel session to discuss DataDish. In email exchanges between this staff (Joanne Spahn) and Eve Essery (CNPP staff), Eve expressed concerns stating:

If this is an IFIC tool, wouldn’t IFIC be presenting it? If you present this, it gives the appearance CNPP/NEL either created or endorses the tool. Am I correct that NEL staff haven’t been informed of this activity and haven’t seen the tool? I feel like we need more information. Is it possible to provide us the draft tool- potentially highlighting what’s different compared to the RDI checklist? (And why was a tool selected that is no longer in use?)

Spahn continued:

Additionally, management voiced concerns that your presentation would imply CNPP endorsement or co-sponsorship with IFIC on the tool.

## Discussion

The study team reviewed emails and documents obtained via public records requests related to IFIC and the IFIC Foundation, with the purpose of describing how IFIC generates and disseminates nutrition information to policy stakeholders and the general public. Results from this content analysis suggest IFIC communicates nutrition information to broad audiences using a variety of tactics designed to shape preferences about the link between unhealthy foods and chronic disease outcomes, manufacture doubt about existing evidence linking certain foods to negative health outcomes, and influence key opinion leaders in academia and government positions to support limited public health interventions designed to reduce consumption of unhealthy foods.

This content analysis extends preliminary findings by Steele and colleagues (2022) who demonstrate connections between IFIC and the food and beverage industry, and how these connections influence IFIC communications about nutrition science [41]. The study by Steele and colleagues (2022) establishes this connection via 75 pages of documents from this repository and point towards the need for analyses of more documents with a focus on how IFIC and its affiliates frame and disseminate their messages [41]. This present study addresses this important research need by collecting, organizing, and analyzing content across multiple years and among a variety of individuals across academia, industry, government, and media.

Results from this study confirm that, at least for one fiscal year, IFIC was funded mostly by large food and beverage companies and the USDA, with the for-profit companies comprising the majority of contributors and many of whom are represented as members of the IFIC Board of Trustees. The extent to which IFIC activities are funded by large food and beverage companies, and the extent to which these companies exert guidance over IFIC activities via board representation, extends prior evidence suggestive of IFIC as an industry front group [4, 6]. Recently, Steele and colleagues (2019) concluded that given the close connection between IFIC and Coca-Cola, it is possible that IFIC promotes industry positions “by stealth.” [6] Sacks and colleagues (2018) previously reviewed email exchanges between former Coca-Cola executives Michael Ernest Knowles and Alex Malaspina, who discuss the value of IFIC as a public relations company with influence over global debates about nutrition and health [4]. In that study, the authors make clear the role that IFIC plays as one of several non-profits, alongside ILSI, to support food and beverage business interests which are often in conflict with broad public health interventions [4]. Specifically, Sacks and colleagues (2018) highlight IFIC goals to critique policy recommendations related to SSB taxes and size restrictions using ties with academic researchers who curate evidence in support of industry positions [4]. Prior to this current study, though, it was not clear how IFIC and its affiliates carried out such activities. Our findings provide examples of how IFIC used close ties with media outlets to anticipate public debate related to food and nutrition – often related to added sugars and ultra-processed foods – and connects these media outlets with researchers who curate a thin subset of evidence in support of industry positions. In doing so, IFIC appeared to act in opposition to its mission of promoting a “global environment where credible science drives food decisions.” [26]

The findings derived from IFIC consumer surveys and the external evidence communicated by IFIC-supported academic researchers consistently focused on individual or “person-level” changes to diet and health. This individualistic narrative is consistent with those promoted by other health harming industries such as the tobacco and alcohol industries [42–44] and prior findings from studies of food and beverage companies [12]. A shift towards systems-level interventions is a crucial component of the public health response to diet-related chronic diseases [45], given increases in the prevalence of type-2 diabetes, obesity, and hypertension [35, 46], and the potential benefits of taxes [47–50] and marketing restrictions [51, 52]. Opposition to these broad interventions is often characterized by narratives of personal responsibility [53–56], which may be traced to political ideologies typically opposed to broad government intervention to reduce consumption of harmful products [42]. There is some evidence that legislatures characterized as having politically-conservative ideologies may be less likely to pass broad obesity-prevention policies, compared to those representing more progressive voters [57, 58]. It therefore follows that promoting a personal choice narrative surrounding diet and health supports the business interests of IFIC funders, and may explain why the talking points provided by IFIC to media outlets are mostly focused on individual decision-making, rather than political interventions. Overall, personal choice narratives like those supported by IFIC bolster food and beverage industry efforts to weaken the regulatory environment in which they operate [59, 60].

The extent to which IFIC’s media strategy actually impacted public perceptions related to nutrition and obesity remains uncertain. However, prior research has established the resilience of individually-framed narratives [53, 54, 56, 61, 62] and scientific misinformation [63] in the public discourse. Based on our review of scientific evidence presented to large media outlets by academic researchers on behalf of IFIC, there is reason to consider IFIC a purveyor of nutrition-based misinformation. Of course, the data we reviewed may insufficiently characterize the content and dissemination strategies of IFIC communications. Further research similar to this current study may provide additional examples of the evidence provided by IFIC affiliates to popular media. Similarly, IFIC may itself wish to provide copies of these media briefings to bring about more transparency related to its role as a scientific communications organization.

Our study is subject to several limitations. First, studies based on public records may be biased based on the interpretations of the study team. To reduce these potential biases, we have provided direct quotes to provide the reader with an opportunity to appraise our conclusions. Additionally, we have only provided evidence that was repeated and unrefuted in the available data, so as to reduce the possibility that our findings are based on abnormal or infrequent events. Finally, the documents we reviewed did not contain data from the last three years, during which IFIC has transitioned to new leadership. It is therefore unclear if these data represent an ongoing pattern of tactics used to promote business-friendly narratives related to diet and health. It is important to conduct future research using documents from more recent years to establish if and how a pattern has continued.

## Conclusions

Our study shows that IFIC uses a combination of tactics to promote specific business-friendly narratives about dietary intake, food safety, and nutrition research. Based on our findings, it appears that IFIC and its affiliates leverage connections with multimedia outlets to pro-actively disseminate counterarguments to emerging research about the potential adverse health impacts of sugar consumption. Additionally, IFIC and its affiliates appear to promote an individualistic narrative of dietary intake and weight gain, thereby shifting the focus of interventions away from politics, systems and structures that predispose certain groups of people to diet-related health disparities. IFIC also produces its own survey research and uses relevant findings to create educational tools that target policymakers and stakeholders at national and international levels. In doing so, IFIC promotes food and beverage company interests and undermines the accurate dissemination of scientific evidence related to diet and health.

## Supplementary Information

**Additional file 1.** Codebook.

**Additional file 2.** Newsletter.

**Additional file 3.** IFIC Total Revenue, Program Services, and Membership Dues, 2004–2018.

**Additional file 4. **Companies solicited for voluntary contribution to IFIC *Understanding Our Food* initative, 2014.

**Additional file 5.** Kern bibliography.

**Additional file 6.** 2012–2015 Strategy.

## Data Availability

U.S. Right to Know collected the data set for this paper, which includes documents obtained via state FOI, federal FOIA and litigation discovery. The documents were donated to the UCSF Industry Documents Library, and will be available at https://www.industrydocuments.ucsf.edu.

## Abbreviations

IFIC: International Food Information Council
USDA: United States Department of Agriculture
SSBs: sugar-sweetened beverages
