# Supplementary material for: How independent is the international food information council from the food and beverage industry? A content analysis of internal industry documents
Source: Global Health. 2022 Oct 29;18:91. doi: 10.1186/s12992-022-00884-8 (PMC9618198; doi:10.1186/s12992-022-00884-8)
Supplement: Supplementary file 1 — Additional file 1. Codebook. [file 12992_2022_884_MOESM1_ESM.docx]

| Code | Full Description | When to Use Codes |
| --- | --- | --- |
| Political Environment |  | Select this grouping of codes when the document mentions the use of political environment as a vehicle of power |
| Lobbying | Lobbying government officials | **Select this code when the document mentions IFIC attempting** to influence the decisions of the government |
| Revolving Door | Revolving door | **Select this code when the document mentions IFIC using** revolving door (defined as the movement of high-level employees from public-sector jobs to private sector jobs and vice versa) |
| Donation | Campaign and party donations | **Select this code when the document mentions IFIC d**onating to political parties or candidates |
| Direct Participation | Direct Participation in Governmental Agencies, Committees, and Commissions, and Partnerships with Government for Policy Delivery | **Select this code when the document mentions IFIC b**ecoming partners in the formulation of public policy |
| Treaties | International Treaties and Trade Agreements | **Select this code when the document mentions IFIC u**sing international treaties/ trade agreements to support industry preferred outcomes |
| Aid | Tied development aid | **Select this code when the document mentions IFIC l**obbying governments to formulate development aid in favor of the industry |
| International Organizations | Pressures on International Organizations | **Select this code when the document mentions IFIC p**rotecting industry interests via their governmental representatives or directly via participation in delegations to international bodies with mandates to regulate their activities. |
| Preference Shaping |  | Select this grouping of codes when the document mentions the use of preference shaping as a vehicle of power |
| Public Relations | Business Associations and PR Companies | **Select this code when the document mentions IFIC m**ounting multi- pronged campaigns that target the media, legislators and consumers to dismiss public health concerns. |
| Foundations and Philanthropy | Corporate Foundations and Philanthropy | **Select this code when the document mentions IFIC u**sing philanthropic institutions as channels for unhealthful corporate products |
| Opinion Leader | Spokespersons and Key Opinion Leaders | **Select this code when the document mentions IFIC** enlisting key opinion leaders to shape the background of accepted issues and opinions in a given field and prepare the target audience to welcome new drugs, technologies and diagnosis. |
| Health Professional Organizations | Health Professional Organizations | **Select this code when the document mentions IFIC funding** health professional organizations |
| Manufacturing Doubt | Manufacturing Doubt | **Select this code when the document mentions IFIC** casting doubt on the scientific evidence documenting negative effects associated with them and discrediting the scientists who produce such evidence. The rationale is that if the existing evidence around the harmful effects of a given product is ambiguous and there is no consensus around it, then there is no need for regulatory action |
| Framing | Corporate Spinning and Framing | **Select this code when the document mentions IFIC f**raming public health issues in terms of personal responsibility for making informed choices takes the onus away from the harmful composition of certain products, from their availability, marketing and advertising and ultimately from their regulation. |
| CSR | Corporate social responsibility | **Select this code when the document mentions IFIC u**sing CSR to increase exposure to harmful corporate products |
| Front Groups | Civil society capture: corporate front groups, think tanks and consumer groups | **Select this code when the document mentions IFIC** co-opting grassroots formations - e.g. consumer and patient groups, or research organisations – e.g. think tanks |
| Manufacture Disease | Manufacture Disease | **Select this code when the document mentions IFIC** creating new diagnostics and thresholds for medical intervention |
| Media | Capture of Media | **Select this code when the document mentions IFIC h**arnessing the power of media to hedge against the coverage of harmful stories and secure favorable treatment of corporate activities and image |
| Marketing | Marketing and Advertising | **Select this code when the document mentions IFIC** using marketing and advertising to expand the number of consumers for a given product and shape the psychological and social predisposition to accept and endorse hyperconsumption. |
| Product Modification | Product Modification and Targeting Vulnerable Populations | **Select this code when the document mentions IFIC e**xpanding to the developing world/untapped market, targeting vulnerable populations, and or modifying the product to increase sales |
| Knowledge Environment |  | Select this grouping of codes when the document mentions the use of knowledge as a vehicle of power |
| Science to specification | Science to Specification | **Select this code when the document mentions IFIC s**etting the research agenda by funding research at universities, in-house corporate laboratories, non-profit research institute or for-profit science-for-hire firms; funding allows control over study design, analysis methodology and ownership of data; suppressing or misreporting unfavorable results through exclusive ownership of the data |
| Funding Medical Education | Funding Medical Education | **Select this code when the document mentions IFIC f**unding of symposia, hospital lectures, and medical specialty meetings ensures that the educational content is shaped to favour certain products and procedures over others, without mandatory scrutiny of claims of superiority. |
| Science Laundromats | Science Laundromats | **Select this code when the document mentions IFIC c**ontrolling image once health damaging effects of corporate products are made public. SABs are organized groups of “industry friendly” “third party” scientists who support industry’s scientific posi- tions in regulatory processes, the courtroom and public opinion |
| Legal Environment |  | Select this grouping of codes when the document mentions the use of legal environment as a vehicle of power |
| Limit liability | Limit liability | **Select this code when the document mentions IFIC** changing the law and reinterpreting its spirit and intent to protect corporate interest |
| Legal Threats | Threat of litigation and pre-emption | **Select this code when the document mentions IFIC** using the possibility of costly and time- consuming litigation to deter action that may bring the public’s attention to unhealthful products and practices. |
| Unregulated Activity | Unregulated Activity | **Select this code when the document mentions IFIC** keeping prices of harmful products artificially low and more available when the final price does not reflect the full cost of production |
| Extra Legal Environment |  | Select this grouping of codes when the document mentions the use of extra legal environment as a vehicle of power |
| Fragmentation | Opposition Fragmentation | **Select this code when the document mentions IFIC** discrediting public health advocates’ opposition or portraying the public health community as fragmented to ensure that there is no countervailing discourse. |
| Illegal | Illegal Activity | **Select this code when the document mentions IFIC** bribing, smuggling and illicit trade, and price fixing |
| Tax Evasion | Tax Evasion | **Select this code when the document mentions IFIC attempting** to evade tax |
| **Adapted from Lima & Galea (2018) ^28^** | | |
