# Supplementary figures and images for: How independent is the international food information council from the food and beverage industry? A content analysis of internal industry documents
Source: Global Health. 2022 Oct 29;18:91. doi: 10.1186/s12992-022-00884-8 (PMC9618198; doi:10.1186/s12992-022-00884-8)

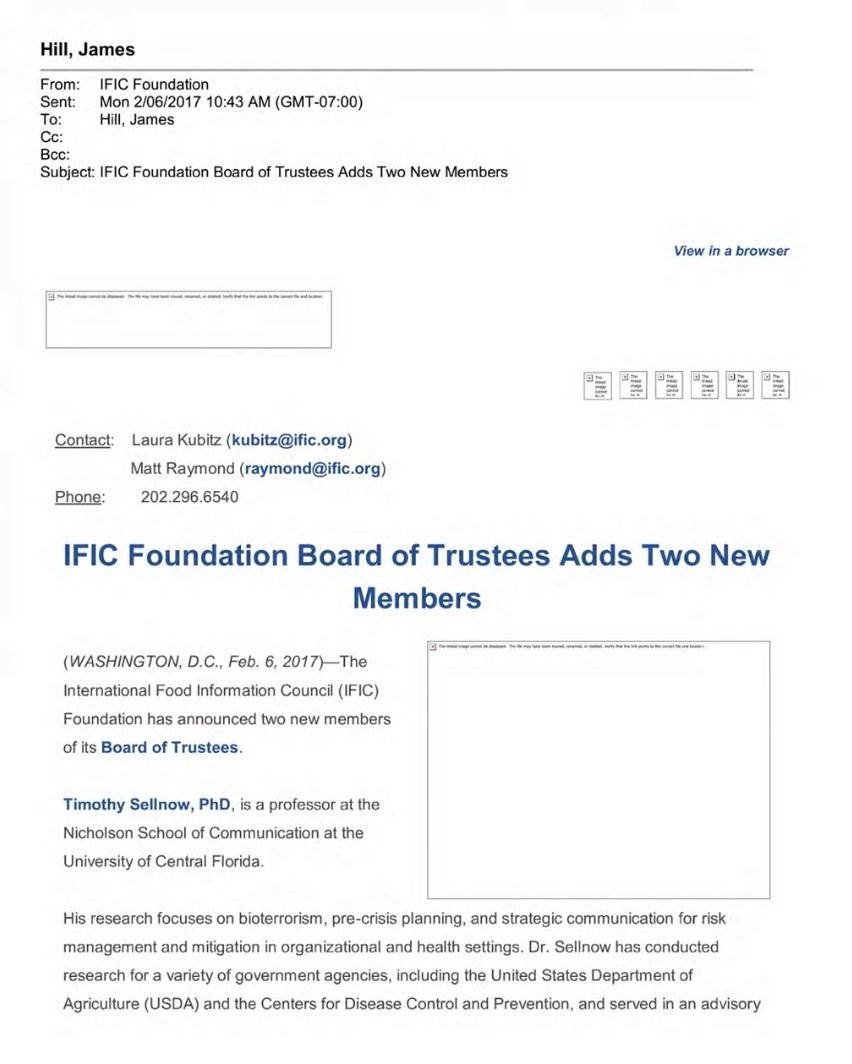

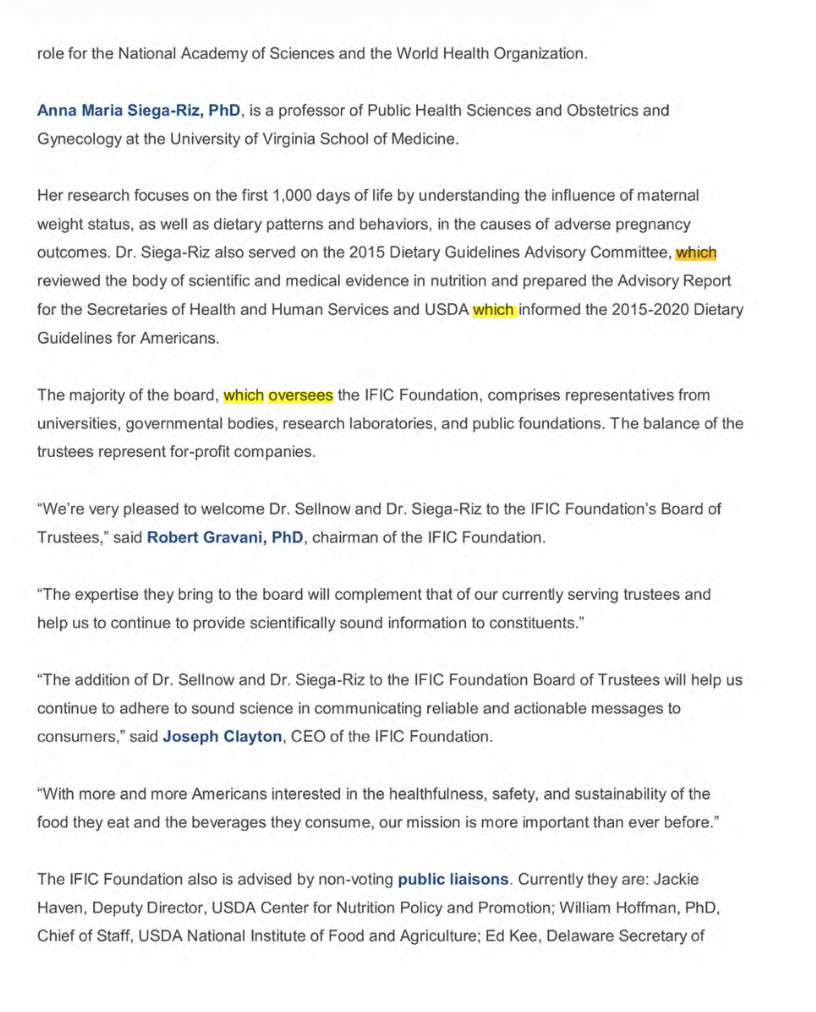

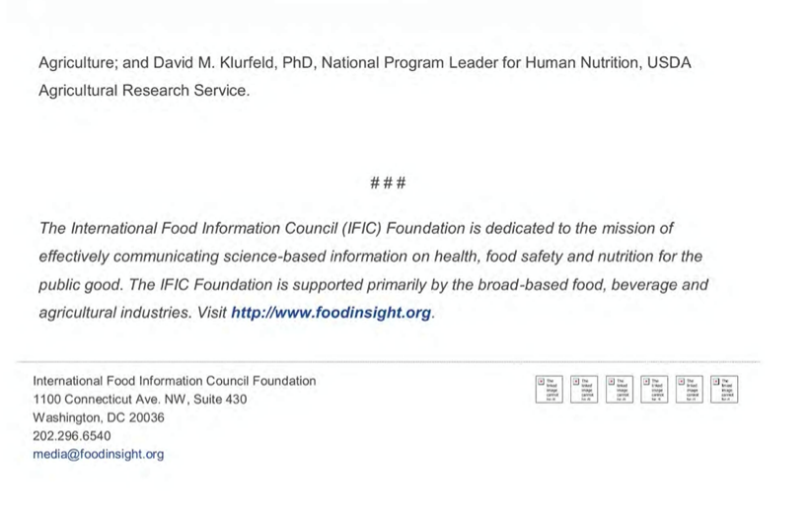

Supplement: Supplementary file 2 — Additional file 2. Newsletter. [file 12992_2022_884_MOESM2_ESM.docx]
