# Supplementary material for: How independent is the international food information council from the food and beverage industry? A content analysis of internal industry documents
Source: Global Health. 2022 Oct 29;18:91. doi: 10.1186/s12992-022-00884-8 (PMC9618198; doi:10.1186/s12992-022-00884-8)
Supplement: Supplementary file 3 — Additional file 3. IFIC Total Revenue, Program Services, and Membership Dues, 2004–2018. [file 12992_2022_884_MOESM3_ESM.docx]

**IFIC Total Revenue, Program Services, and Membership Dues, 2004 - 2018**

| Year | Total Revenue | Total Program Services | Total Membership Dues | Percentage membership dues of total revenue |
| --- | --- | --- | --- | --- |
| 2018 | 3,398,328 | 3,016,048 | 2,969,061 | 87.4% |
| 2017 | 3,654,463 | 3,223,556 | 3,195,350 | 87.4% |
| 2016 | 3,840,843 | 3,330,127 | 3,294,200 | 85.8% |
| 2015 | 5,039,326 | 4,274,106 | 4,126,783 | 81.9% |
| 2014 | 4,958,670 | 4,260,441 | 4,136,783 | 83.4% |
| 2013 | 5,166,092 | 4,459,599 | 4,379,572 | 84.8% |
| 2012 | 4,918,927 | 4,107,688 | 4,042,037 | 82.2% |
| 2011 | 4,780,723 | 4,128,250 | 4,090,075 | 85.6% |
| 2010 | 4,801,665 | 4,359,033 | 4,323,431 | 90.0% |
| 2009 | 4,682,991 | 4,136,842 | 4,101,767 | 87.6% |
| 2008 | 5,200,682 | 4,567,450 | 4,537,550 | 87.2% |
| 2007 | 5,507,509 | 4,999,050 | 4,920,200 | 89.3% |
| 2006 | 4,861,522 | 4,192,293 | 4,235,243 | 87.1% |
| 2005 | 4,938,503 | 4,116,845 | 4,162,590 | 84.3% |
| 2004 | 4,423,217 | 3,728,150 | 3,780,823 | 85.5% |
