# Supplementary material for: How independent is the international food information council from the food and beverage industry? A content analysis of internal industry documents
Source: Global Health. 2022 Oct 29;18:91. doi: 10.1186/s12992-022-00884-8 (PMC9618198; doi:10.1186/s12992-022-00884-8)
Supplement: Supplementary file 4 — Additional file 4. Companies solicited for voluntary contribution to IFIC Understanding Our Food initative, 2014. [file 12992_2022_884_MOESM4_ESM.docx]

**Companies solicited for voluntary contribution to IFIC *Understanding Our Food* initiative, 2014**

| **Company** |
| --- |
| Abbott Nutrition |
| Ajinomoto North America |
| Bayer CropScience |
| Cargill |
| Coca-Cola Company |
| Dannon |
| Dow AgroSciences |
| Ferrero |
| Hershey |
| Kraft |
| Mars |
| McDonalds |
| Monsanto |
| Nestle |
| PepsiCo |
| Red Bull |
| J.M. Smucker |
| Dupont |
| Zoetis |
