# Supplementary material for: How independent is the international food information council from the food and beverage industry? A content analysis of internal industry documents
Source: Global Health. 2022 Oct 29;18:91. doi: 10.1186/s12992-022-00884-8 (PMC9618198; doi:10.1186/s12992-022-00884-8)
Supplement: Supplementary file 5 — Additional file 5. Kern bibliography. [file 12992_2022_884_MOESM5_ESM.docx]

| Title | Authors | Year | Journal | Study question | Study design | Animal | Sample size | Author conclusions | Funding |
| --- | --- | --- | --- | --- | --- | --- | --- | --- | --- |
| Dietary sugars stimulate fatty acid synthesis in adults | Parks EJ, Skokan LE, Timlin MT, Dingfelder CS | 2008 | The Journal of Nutrition | "The goal of this study was to determine the magnitude by which acute consumption of fructose in a morning bolus would stimulate lipogenesis (measured by infusion of 13C1-acetate and analysis by GC-MS) immediately and after a subsequent meal." | RCT | No | 6 | "...evidence that fructose causes hyperlipidemia postprandially both directly, through the synthesis of fatty acids, and indirectly, by increasing liver reesterification of fatty acids from all sources" | NIH; Cargill Higher Education Fund; Sugar Association |
| Metabolic effects of dietary fructose in healthy subjects | Swanson JE, Laine DC, Thomas W, Bantle JP | 1992 | American Journal of Clinical Nutrition | "To determine if dietary fructose causes adverse metabolic effects" | RCT | No | 14 | "Thus, our data indicate that dietary fructose has no important long-term effect on fasting or postprandial serum triglycerides in healthy individuals." | International Fructose Association; NIH |
| Effects of dietary fructose on plasma lipids in healthy subjects | Bantle JP, Raatz SK, Thomas W, Georgopoulos A | 2000 | American Journal of Clinical Nutrition | " to determine the effect of dietary fructose on plasma lipids" | RCT | No | 24 | "Dietary fructose was associated with increased fasting and postprandial plasma triacylglycerol concentrations in men. Diets high in added fructose may be undesirable, particularly for men. Glucose may be a suitable replacement sugar" | NIH |
| Blood lipid distribution of hyperinsulinemic men consuming three levels of fructose | Hallfrisch J, Reiser S, Prather ES | 1983 | American Journal of Clinical Nutrition | "...this study was conducted to determine the effects of three moderate levels of fructose consumed in a normal American diet on blood lipids and blood pressure in normal and hyperinsulinemic males." | RCT | No | 12 | "These results indicate that fructose, fed in moderate amounts in a normal American diet, can produce undesirable changes in blood lipids that are associated with heart disease. Hyperinsulinemic men are more susceptible to these changes than controls." | NA |
| Effects of various levels of dietary fructose on blood lipids of rats | Benado M, Alcantara C, de la Rosa R, Ambrose M, Mosier K, Kern M | 2004 | Nutrition Research | "The focus of our research was to determine the effects of “typical” American consumption patterns of fructose versus a dietvery high in fructose and a diet devoid of fructose on serum lipid levels in rats. " | RCT | Yes | 40 | "...this study demonstrates in an animal model that the potential adverse effects of dietary fructose are minimal under conditions that reflect “average”(10% of energy)American intake, whereas intake approximating just above the 90th percentile of intake (20% of energy) may be detrimental." | NA |
| Fructose ingestion: dose-dependent responses in health research | Livesey G | 2009 | The Journal of Nutrition | NA | Commentary | NA | NA | NA | Dansico Sweeteners; ILSI |
| Fructose consumption and consequences for glycation, plasma triacylglycerol, and body weight: meta-analyses and meta-regression models of intervention studies | Livesey G, Taylor R | 2008 | American Journal of Clinical Nutrition | "The glycemic response to dietary fructose is low, which may improve concentrations of glycated hemoglobin (HbA1c, a marker of dysglycemia). Meanwhile, adverse effects on plasma triacylglycerol (a marker of dyslipidemia) and body weight have been questioned. Such effects are reported inconsistently. We aimed to evaluate the effect of fructose on these health markers, particularly exam" | SRMA | NA | 42 | "The meta-analysis shows that fructose intakes from 0 to 90 g/d have a beneficial effect on HbA1c" | Danisco Sweeteners |
| Twenty-four hour endocrine and metabolic profiles following consumption of high fructose corn syrup-, sucrose-, fructose-, and glucose-sweetened beverages with meals | Stanhope KL, Griffen SC, Bair BR, Swarbrick MM, Keim NL, Havel PJ | 2008 | American Journal of Clinical Nutrition | " the objectives of this study were to compare the metabolic and endocrine effects of consuming HFCS- and sucrose-sweetened beverages, and to determine if responses are affected by gender and adiposity" | RCT | No | 34 | "Sucrose and HFCS do not have substantially different short-term endocrine/metabolic effects." | PepsiCo |
| Honey promotes lower weight gain, adiposity, and triglycerides than sucrose in rats | Nemoseck TM, Carmody EG, Furchner-Evanson A, Gleason M, Li A, Potter H, Rezende LM, Lane KJ, Kern M | 2011 | Nutrition Research | "The purpose of this study was to compare the influences of feeding a honey-based diet vs a sucrose-based diet for 33 days on weight regulation, adiposity and related biomarkers, and lipid metabolism in male Sprague-Dawley rats." | RCT | Yes | 36 | "the results suggest that there may be numerous promising health benefits, such as improved weight regulation and reduced triglyceride levels, when sucrose is substituted with honey in the diet; however, non–HDL cholesterol was higher for honey-fed rats, which was against our hypothesis." | National Honey Board |
| Type of snack influences satiety responses in adult women | Furchner-Evanson A, Petrisko Y, Howarth L, Nemoseck T, Kern M | 2010 | Appetite | "The objective of the current study was to assess the responses of two snack choices similar in fat, protein, carbohydrate, and sugar contents while differing in fiber content, on satiety, subsequent food intake, and plasma glucose, insulin, and ghrelin responses" | Pre/post | No | 21 | "Results of this study indicate that a whole fruit snack of dried plums promotes greater satiety than low-fat cookies, but that food consumption 2 h after snack intake are not significantly affected." | California Dried Plum Board |
| Snack selection influences nutrient intake, triglycerides, and bowel habits of adult women: a pilot study | Howarth L, Petrisko Y, Furchner-Evanson A, Nemoseck T, Kern M | 2010 | Journal of the American Dietetic Association | "This research was designed to determine the effects of consumption of a whole snack food (dried plums) vs a common, commercially available, carbohydrate-rich, snack food (low-fat cookies) as between-meal feedings in adult American women on daily energy consumption, nutrient intake, and metabolic responses." | RCT | No | 29 | "Because dried plums are rich in nondigestible carbohydrates, antioxidants, and other nutrients, their use as a snack food may be beneficial. Our results suggest that this can improve overall dietary intake and promote positive bowel responses without the adverse effects on lipid concentrations that can occur with consumption of other carbohydrate-rich snacks." | California Dried Plum Board |
| Sugar-added beverages and adolescent weight change | Berkey CS, Rockett HR, Field AE, Gillman MW, Colditz GA | 2004 | Obesity Research | "we analyzed the relationship between intakes of beverages (milk, sugar-added beverages, fruit juices, and diet soda) and changes over time in BMI" | Longitudinal | No | >10,000 | "Data from our cohort suggested that children who reduce intakes of sugar-added beverages, along with other behavior modifications such as increasing physical activity and reducing time with TV/videos/computer games, may prevent excessive weight gains that can lead to obesity." | NIH; Boston Obesity Nutrition Research Center; CDC; USDA; Kellogs |
