# Supplementary material for: How independent is the international food information council from the food and beverage industry? A content analysis of internal industry documents
Source: Global Health. 2022 Oct 29;18:91. doi: 10.1186/s12992-022-00884-8 (PMC9618198; doi:10.1186/s12992-022-00884-8)
Supplement: Supplementary file 6 — Additional file 6. 2012–2015 Strategy. [file 12992_2022_884_MOESM6_ESM.docx]

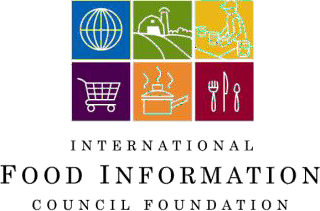


INTERNATIONAL FOOD INFORMATION COUNCIL FOUNDATION

2012-2015 STRATEGY

VISION: A global environment where credible science drives food policy and consumer choice.

MISSION: To effectively communicate science-based information on health, nutrition and food safety for the public good.

OVERARCHING GOAL: Advance public knowledge and translate the science of nutrition, food safety and health in an appealing and credible way, with increased support from stakeholder partners.

GOALS:

# The Foundation will help IFIC implement its strategy by successfully achieving its educational mission outlined in the IFIC strategy: “Clear, Credible, Insightful.”

Objective

Work synergistically with IFIC committees and programs to achieve action plan within budget as outlined in “Clear, Credible, Insightful”

# The Foundation will be recognized by key stakeholders as a credible convener on consumer attitudes and behavior related to food safety and nutrition and their role in health promotion and disease risk reduction, including non-communicable diseases.

Objectives

- 1. Increase awareness of IFIC Foundation’s leadership role in behavioral nutrition and health
  2. Advance visibility and effectiveness of International Center of Excellence in Food Risk Communication
  3. Elevate value and significant insights of the *Food and Health Survey* to better inform strategic initiatives with behavioral focus

# Public dialogue on modern food production will improve as a result of the Foundation’s leadership and educational outreach to key stakeholders.

Objectives

- 1. Expand awareness and use of *Understanding our Food* toolkit with all stakeholders
  2. Repurpose *Understanding our Food* toolkit for elementary school supplemental curricula
  3. Engage media and other key stakeholders in collaborative dialogue to advance understanding of food production and its relation to health, while clarifying public misperceptions and inconsistencies in the literature

# The Foundation will expand its base of funding support from sources external to IFIC members to at least 25% by 2015.

Objectives

- 1. Increase revenue by earning market value of educational resources
  2. Determine need for additional development resources by 2013
  3. Seek government and other outside grant/revenue developing partnerships/programs consistent with the Foundation mission
